# Personal

**Published:** 1899-08

**Authors:** 


### PERSONAL.

DR. WALTER BLACKBURN DORSETT, of St. Louis, was elected president of the Missouri State Medical Association at its forty-second annual meeting, held at Sedalia, May 16-18, 1899. Commenting upon the fitness of this choice by the association the *Medical Herald* for June appropriately says :

After a preparatory education at Washington University, Dr. Dorsett began to study medicine in 1875, under Dr. LeGrand Atwood; attended St. Louis Medical College and was graduated March 14, 1878. He was assistant surgeon to St. Louis City Hospital, 1878-79; dispensary physician city health department, 1879-87; superintendent and surgeon-in-charge of St. Louis Female Hospital, 1887-92; is professor of obstetrics in Beaumont Hospital Medical College; gynecologist to the German Evangelical Deaconess's Hospital, and gynecologist on the staff of the Missouri Baptist Sanitarium.

Dr. Dorsett is a member of the St. Louis Medical Society and was its president in 1892; St. Louis Obstetrical and Gynecological Society, of which he was secretary in 1893-94, and at the present is its president; St. Louis Surgical Society Missouri State Medical Association; American Association of Obstetricians and Gynecologists, its second vice-president, 1897-98; City Hospital Medical Society of St. Louis.

He is a frequent contributor to the medical press, and possesses a genial disposition, which makes him a warm friend to his confréres.

---

Dr. Edward N. Brush, superintendent of the Sheppard and Enoch Pratt hospital for the Insane, has been appointed professor of psychiatry at the College of Physicians and Surgeons, Baltimore. Dr. Brush brings an ample equipment to his new chair, being by experience and training one of the most accomplished alienists in this country and his many friends in Buffalo, where he formerly resided, are especially gratified to learn of this further recognition of his talents in the chosen field of his work.

---

Dr. John McG. Woodbury, of New York, lately chief division surgeon of volunteers, now clinical professor of orthopedic surgery in the medical department of Cornell University, has been appointed a commissioner on behalf of the United States for the purpose of observing and reporting on the medico-military organisation of the German army in active operation during the fall maneuvres.

---

Drs. Joseph M. Mathews and L. S. McMurtry, of Louisville, and Dr. I. N. Love, of St. Louis, paid a short visit to Buffalo, July 8, 1899, when they were entertained at breakfast by Dr. William Warren Potter. Afterward the distinguished tourists went to Niagara Falls for a few days of rest and recreation.

DR. FRANK WHITEHILL HINKEL, of Buffalo, sailed for Europe, July 27, 1899, on the North German Lloyd steamer, Barbarossa. He will attend the Triennial International Otological Congress, at London, August 8-12, and afterward spend some time in recreation. He expects to return about September 15th.

DR. RUFUS BARTLETT HALL, of Cincinnati, was elected president of the Ohio State Medical Society at its annual meeting, held in Springfield, May, 1899.

